# Posterolateral transpedicular approach for ventrally seated cervicothoracic spinal cord lesions: how I do it

**DOI:** 10.1007/s00701-022-05424-2

**Published:** 2022-11-22

**Authors:** P. M. F. Cristaldi, A. Parlangeli, D. Nicoli, M. Incerti

**Affiliations:** 1 Department of Medicine and Surgery, School of Medicine, University of Milano-Bicocca, Milan, Italy; 2 Unit of Neurosurgery, Polyclinic of Monza, Monza, Monza-Brianza, Italy

**Keywords:** Transpedicular approach, Ventral spinal lesion, Spinal cord tumor, Surgical approach, Cervicothoracic region

## Abstract

**Background:**

Surgical exposure of lower cervical and upper thoracic intradural extramedullary lesions located along the ventral medulla are among the most complexes to address in spinal surgery, and their surgical removal carries a high risk.

**Methods:**

We describe the surgical steps of a posterolateral transpedicular approach for resection of an intradural extramedullary lesion located anterolaterally at C7-T1 level.

**Conclusions:**

A posterolateral transpedicular approach is a safe and efficient surgical corridor to explore the ventral spinal cord and to have a direct access to lower cervical-upper thoracic lesions without the extensive manipulation of the spinal cord and the spine instability.

**Supplementary Information:**

The online version contains supplementary material available at 10.1007/s00701-022-05424-2.

## Introduction

Ventrally seated cervical and thoracic lesions are removed through different surgical approaches. The surgical choice principally depends upon the location and size of the tumor as well as its characteristics, the spinal stability, the control of the spinal cord (SC), and blood vessels [1, 4]. Two principal surgical approaches to access this region have been described, with varying degrees of exposure and approach-related morbidity: anterior corpectomy with instrumented fusion and posterior or posterolateral approaches [1–3].

## Relevant surgery anatomy

Several facets of cervical and thoracic spine anatomy bear relevance to surgical practice. The bony anatomy (pedicles, lamina, transverse process, lateral mass, facet joints, and spinous process), the cervicothoracic junction, the ligamentous structures, and the muscle layers must be in-depth known. Stability is provided by a complex combination of osseous, muscular, and ligamentous supports. The posterior cervical musculature can be divided into three main groups from superficial to deep. The semispinalis cervicis is part of the deepest and is considered the most important posterior stabilizer [5].

## Description of the technique

**Fig. 1:**
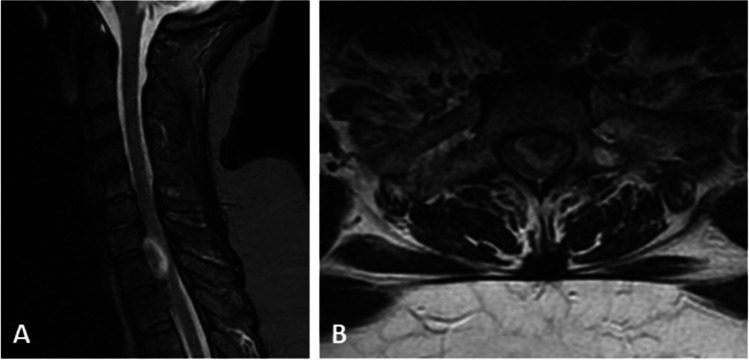
MRI (**a** sagittal sequence on STIR; **b** axial sequence on T2) showed intradural extramedullary lesion located ventrally and on the left side with severe spinal cord compression at the level of C7-T1

We describe in a stepwise fashion a posterolateral transpedicular approach for the resection of an intradural extramedullary lesion left-ventrally located causing a severe SC compression at C7-T1 level (Fig. 1). By this approach, the preservation of contralateral muscle and bony structures may help to avoid a destabilization of the spine integrity.

### Patient preparation

The patient is under general anesthesia and in prone position on a spine table. Somatosensory and transcranial motor evoked potentials (SEPs and MEPs) and free-run electromyography (EMG) are monitored. Standard arthroscopic facilities and conventional spine instruments are used. Fluoroscopic confirmation of the surgical level is made with a needle inserted at the target area.

### Skin incision and muscle dissection

A C-shaped incision is made beginning at C5 until T2 level, with a left-sided convexity at C7-T1. The skin is elevated, and a dissection of the cervical fascia and the trapezius muscle is made for further retraction of the skin flap. The cervical fascia is opened in a C-fashion too. Then, a cervical muscle splitting blunt dissection is made by detaching the muscles from the laminae, starting from the midline until the exposure of the laminae and the joint masses from C7 to T2 on the left side. By this procedure, nuchal muscles are strongly reflected on the left side, preserving at the same time those on the right side. An effective muscle reflection without crushing injury from overretraction is obtained thanks to the C-shaped opening of the skin and cervical muscles.

### Laminotomy, facetectomy, and pediculotomy

Under the microscope, a partial hemilaminotomy until the inferior edge of C7 and the superior edge of T2 and a complete hemilaminotomy of T1 on the left-sided is made with the use of high-speed diamond burrs and Kerrison rongeurs. With a blunt hook dissector, the plane between the ligamentum flavum and the dura is identified. Once exposed, the ligamentum flavum is detached from the laminae and the dural sac is exposed. A partial facetectomy of C7-T1 articular process followed by the undercutting of the pedicle of T1 is performed with the use of rongeurs and a high-speed drill. In this way, the lateral and left-ventral part of the dural sac is well exposed without destroying any bony or muscular structures which might have destabilized the spine integrity.

### Dural incision, tumor resection, and closure

**Fig. 2:**
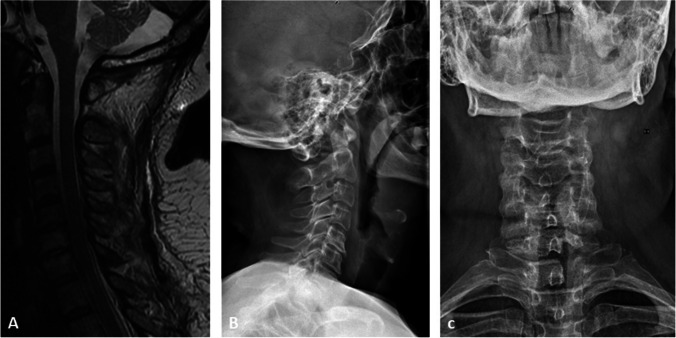
A sagittal MRI T2 sequence (**a**) and a lateral (**b**) and anteroposterior (**c**) cervical radiography performed at 3 and 6 months after surgery, respectively, showed a preservation of the spinal alignment

An incision of the lateral part of the dura matter is made at the point where the tumor is projected. Dural flaps are tacked up with 5–0 prolene suture. The dentate ligament is cut to facilitate the gentle rotation of the SC. An intradural extramedullary lesion that appeared as a firm gray mass is identified. A careful detachment is performed with protection of nerve roots and SC abutting the lesion. Using micro-instrumentations, as well as adjusting the angulation of the spine table and the microscope, the tumor is removed in two fragments. After the resection, dural flaps are approximated meticulously with sutures and reinforced using fibrin sealants to reduce the risk of cerebral spinal fluid (CSF) leakage. No CSF leak is noted upon Valsalva maneuver. No SEP and MEP changes are noted during surgery. A surgical drain is inserted and kept for 24 h after surgery. This approach is involving anatomical structures important for spinal stability, but the preservation of contralateral structures might be helpful to preserve stability. Spinal alignment is preserved, as noticed at the spinal MRI and at the cervical radiography (Fig. 2) done at 3 and 6 months after surgery, respectively. Histologic examination revealed a schwannoma.

## Indications

Intradural extramedullary lesions affect the ventral part of the SC and with a lateral projection. The suitability of this approach for other ventral intradural pathologies (e.g., spinal cord herniations, arachnoid, or neuroenteric cysts) needs to be clarified [3].

## Limitations

1. Lesion should have a lateral projection.
2. Tumor resection alone is not adequate for unstable spine as it requires instrumentation for stabilization.
3. This approach is not suitable for anterior-bilateral lesions: removal of two facet joints may lead to spinal instability.

## How to avoid complications

1. Monitor the SEPs, MEPs, and free-run EMG.
2. Partial facetectomy and pediculotomy and preservation of paravertebral muscles of the contralateral side were performed to prevent cervical instability.
3. Use cotton patties and saline irrigation to avoid dural injuries when drilling.
4. An adequate closure technique was used to prevent CSF leakage.

## Specific information for the patient

1. There is a risk of cervical axial symptoms such as pain and stiffness of the neck due to the posterior approach, as well as other risks regarding the surgical procedure (e.g., numbness over the dermatome of the nerve operated on, SC injuries, and CSF leak).
2. Patients should be aware of the risk of a spinal instability, secondary to the anterior tilt of the cervical spine resulting from a loss of static and functional posterior stabilizing forces. A muscle balance physiotherapy-staged regimen is recommended following surgery as well as a clinical and radiological follow-up.

## Supplementary Information

Below is the link to the electronic supplementary material.

Supplementary file1 (MP4 215425 KB)
